# Increased hepatic and circulating chemokine and osteopontin expression occurs early in human NAFLD development

**DOI:** 10.1371/journal.pone.0236353

**Published:** 2020-07-30

**Authors:** Michael Kriss, Lucy Golden-Mason, Jeffrey Kaplan, Faridoddin Mirshahi, V. Wendy Setiawan, Arun J. Sanyal, Hugo R. Rosen

**Affiliations:** 1 Division of Gastroenterology & Hepatology, Department of Medicine, University of Colorado School of Medicine, Aurora, CO, United States of America; 2 GI and Liver Innate Immune Program, University of Colorado School of Medicine, Aurora, CO, United States of America; 3 Department of Medicine, University of Southern California (USC) Keck School of Medicine, Los Angeles, CA, United States of America; 4 USC Research Center for Liver Disease (RCLD), Los Angeles, CA, United States of America; 5 Department of Pathology, University of Colorado School of Medicine, Aurora, CO, United States of America; 6 Department of Internal Medicine, Virginia Commonwealth University, Richmond, VA, United States of America; 7 Department of Preventive Medicine, University of Southern California (USC) Keck School of Medicine, Los Angeles, CA, United States of America; Medizinische Fakultat der RWTH Aachen, GERMANY

## Abstract

**Background & aims:**

Non-alcoholic steatohepatitis (NASH), a subtype of non-alcoholic fatty liver disease (NAFLD) that can lead to fibrosis, cirrhosis, and hepatocellular carcinoma, is characterized by hepatic inflammation. Despite evolving therapies aimed to ameliorate inflammation in NASH, the transcriptional changes that lead to inflammation progression in NAFLD remain poorly understood. The aim of this pilot study was to define transcriptional changes in early, non-fibrotic NAFLD using two independent biopsy-proven NAFLD cohorts.

**Methods:**

We extracted RNA from liver tissue of 40 patients with biopsy-proven NAFLD based on NAFLD Activity Score (NAS) (23 patients with NAS ≤3, 17 with NAS ≥5) and 21 healthy controls, and we compared changes in expression of 594 genes involved in innate immune function. Using plasma from an independent cohort of 67 patients with NAFLD and 15 healthy controls, we validated the gene changes observed using a multiplex protein assay.

**Results:**

Compared to healthy controls, NAFLD patients with NAS ≥5 had differential expression of 211 genes, while those with NAS ≤3 had differential expression of only 14 genes. Notably, osteopontin (SPP1) (3.74-fold in NAS ≤3, 8.28-fold in NAS ≥5) and CXCL10 (2.27-fold in NAS ≤3, 8.28-fold in NAS ≥5) gene expression were significantly upregulated with histologic progression of NAFLD. Plasma osteopontin (SPP1) and CXCL10 are significantly increased in the presence of NAFLD, regardless of histologic grade. In addition, the plasma levels of these two proteins distinguish clearly between the presence or absence of NAFLD (AUC>0.90).

**Conclusions:**

Osteopontin (SPP1) and CXCL10 are upregulated early in non-fibrotic NAFLD and may serve as valuable non-invasive biomarkers.

## Introduction

Non-alcoholic fatty liver disease (NAFLD) is the most common cause of chronic liver disease in modern society [1–3], representing a spectrum of pathologies, sometimes progressing to cirrhosis and hepatocellular carcinoma [4,5]. Nonalcoholic steatohepatitis (NASH) is the main diagnostic subtype of NAFLD that can predispose patients to advanced fibrosis and liver-related complications [6]. Inflammation develops when the influx of fatty acids into the liver overwhelms physiologically adaptive mechanisms, with ensuing reactive oxygen formation, ER stress, and hepatocellular dysfunction and injury in a process termed lipotoxicity [7]. Although the stage of fibrosis is the best predictor of clinically relevant outcomes in NASH [8], therapeutic trials have primarily focused on improvement in steatohepatitis as defined by the NAFLD Activity Score (NAS) [3]. Indeed, the AASLD guidelines advocate for medical treatment for the subset with biopsy-proven NASH [9]. Similarly, the EASL guidelines recommend pharmacotherapy for patients with progressive NASH (bridging fibrosis and cirrhosis), as well as for early-stage NASH with high inflammatory activity and/or those with increased risk of fibrosis progression (i.e., age >50 years; diabetes, metabolic syndrome, increased ALT) [10].

In the current study, we sought to identify which transcriptional patterns were upregulated in the livers of subjects with mild (NAS ≤ 3) versus advanced (NAS ≥ 5) who had minimal fibrosis. Furthermore, in an independent cohort of 70 biopsy-proven cases of NAFLD, we sought to determine whether proteins encoded by these genes correlated with the severity of NAFLD.

## Methods

### Patients

We retrospectively identified patients who had undergone liver biopsy at University of Colorado Hospital from 2011–2017 and had a histologic diagnosis of non-fibrotic NAFLD. The NAFLD activity score (NAS) was used to score histologic activity [11].Fibrosis was assessed using NASH CRN classification (11) and we defined non-fibrotic patients as those with no fibrosis or stage 1 fibrosis based on NASH CRN scoring system (includes 1A, 1B, or 1C). Cases were then retrospectively reviewed by a single transplant hepatologist (M.K.) to ensure clinical diagnosis was consistent with NAFLD and any case with Electronic Medical Record (EMR) documentation of significant alcohol use (>1 drink/day for women, >2 drinks/day for men) or additional chronic liver disease diagnosis based on medical history, laboratory testing, or medication use was excluded. Healthy controls were retrospectively identified from patients who had undergone liver biopsy as part of pre-donation biopsy for living liver donation with complete serologic testing for chronic liver disease that was negative, no significant alcohol use prior to evaluation (>1 drink/day for women, >2 drinks/day for men), and histologically normal livers, notably without evidence of steatosis, inflammation, or fibrosis.

### Clinical phenotyping

Demographic, clinical, and laboratory data were collected for each patient enrolled. Demographic data included age, sex, and ethnicity. Clinical risk factors for NAFLD were identified via chart review and included: body mass index (BMI), diabetes mellitus (± long term insulin use), hypertension (defined as high blood pressure at time of biopsy or prescribed anti-hypertensive), hyperlipidemia (± lipid lowering therapy defined as HMG-CoA reductase inhibitor or fibrate), obstructive sleep apnea (OSA), polycystic ovarian syndrome (PCOS), hypothyroidism (defined as elevated thyroid stimulating hormone or prescribed thyroid hormone replacement), and history of cardiovascular disease. Laboratory data included aspartate aminotransferase (AST), alanine aminotransferase (ALT), alkaline phosphatase (AP), total bilirubin (TB), albumin (Alb), and total protein (TP). All controls had routine donor testing that included serologic testing for metabolic risk factors (HgA1c, TSH, fasting lipids), chronic viral hepatitis, autoimmune liver disease, iron overload, and Wilson’s disease per evaluation protocol.

### Histologic evaluation

Formalin-fixed, paraffin-embedded (FFPE) liver tissue sections were stained with hematoxylin and eosin stain and trichrome stain, respectively. All biopsies were read by one of three pathologists, all with subspecialty training in gastrointestinal and liver pathology. All patients had 5% or greater steatosis present on biopsy. The NAFLD Activity Score (NAS) was used to divide subjects into two cohorts: NAS ≤3 (consistent with “probably not” or “borderline” NASH) versus NAS ≥5 (consistent with “probably” or “definite” NASH). Fibrosis was assessed using the NASH CRN classification system.

### RNA extraction from FFPE liver tissue

Two to four curls from continuous FFPE tissue sections per specimen were collected in 1.5mL centrifuge tubes used for RNA isolation. The tubes containing the tissue curls were deparaffinized following the protocol for tube-sections in the Roche HighPure FFPET RNA Isolation spin-column kit (Catalog #06650775001) with the following modifications: maximum 10μM sections used were increased up to 4 curls rather than 1 per tube and d-limonene (histology grade) was used in place of xylene. The tissue sections were allowed to dry completely before proceeding to the RNA Isolation protocol. RNA isolation was performed using the same Roche HighPure FFPET RNA Isolation spin-column kit according to manufacturer’s specifications. RNA was quantified using the NanoDrop 1000 Spectrophotometer (Thermo Fisher Scientific), and a sample subset was additionally quality checked using the High Sensitivity RNA assay with the Qubit 2.0 Fluorometer (Invitrogen). All samples were additionally run using the High Sensitivity RNA Analysis Kit on a 5200 Fragment Analyzer Automated CE System (Agilent) to assess quality and determine nCounter assay input.

### NanoString nCounter^®^ system processing and analysis

Depending on RNA integrity, 300-350ng of the purified RNA was hybridized with the Human Immunology v2 Code Set (NanoString Technologies) using the XT protocol at 65C for 19hours. Samples were cooled to 4°C at completion of hybridization time cycle. Further purification and binding of the hybridized probes to the optical cartridge was performed on the nCounter Prep Station using high sensitivity settings, and finally the cartridge was scanned on the nCounter Digital Analyzer. Raw counts from each gene were imported into the nSolver Analysis Software and normalized against background and housekeeping genes, and overall assay performance was assessed through evaluation of built-in positive controls. A total of 594 immunology-related human genes and 15 internal reference genes were implemented in the digital transcript counting (nCounter^®^ Human Immunology Panel v2 kit assay NanoString^®^, Seattle, WA). Data were analyzed using the nSolver Analysis Software v2.6 gene expression analysis module (NanoString^®^).

### Plasma samples

Plasma was obtained from an independent cohort of subjects with NAFLD (n = 67) and healthy controls (n = 15). NAFLD activity score (NAS) was assessed on liver biopsy by an independent pathologist. Subjects with fatty liver disease were grouped according to their NAS scores (0–1 n = 8, 2 n = 10, 3 n = 22, 4 n = 14, 5 n = 8, 6 n = 8). The study was approved by the Colorado Multiple Institution Review Board and the Virginia Commonwealth University Institutional Review Board.

### Multiplex ELISA

Plasma proteins were assayed using a R&D Systems 5-Plex Luminex® Human Magnetic Assay Kit (CCL20/MIP-3 alpha, CCL22/MDC, CXCL10/IP-10/CRG-2, IL-8/CXCL8, Osteopontin/OPN) according to the manufacturers protocol. All samples were assayed in triplicate and using Luminex xMAP® technology and analyzed using a 5-parameter logistic regression model.

### Statistical analysis

Clinical data was described using mean ± standard deviation (normal distribution) and median [interquartile range (IQR)] (non-normal distribution). Differences in clinical variables and gene expression between groups was analyzed using Chi-square or Fischer’s exact test for categorical variables and Kruskal-Wallis test for continuous variables. Multiple comparisons were accounted for using Dunn’s test. For gene expression analysis, coefficient of variation was used to exclude outliers and differences between groups were determined using one-way ANOVA and Welch’s t-test. Multiple comparisons were accounted for using Benjamini-Yekutieli False Discovery Rate. Spearman’s correlation coefficient was used to analyze the relationship between clinical variables and gene expression. For plasma protein analysis, a two-tailed unpaired t test was used to compare the differences between groups. A p-value of 0.05 or less was considered significant. To investigate the diagnostic accuracy of OPN and CXCL10 in the prediction of NAFLD, we used receiver operating characteristic (ROC) regression to calculate the area under the ROC curve for OPN and CXCL10 separately and in combination. Models were adjusted for age and sex. ROC analysis was conducted using SAS 9.4.

## Results

### Clinico-pathological characteristics of study cohort

A total of 40 cases with NAFLD were identified, 23 with NAS ≤ 3 and 17 with NAS ≥ 5, as well as 21 healthy controls. The mean NAS score in the NAS ≤3 group was 3.0 ± 0.2 vs 5.5 ± 0.5 in the NAS ≥5 cohort (p<0.001). Importantly, only 3/23 (13%) patients in the NAS ≤ 3 cohort met criteria for NAFL (bland steatosis without ballooning degeneration and no/minimal lobular inflammation) while all patients in the NAS ≥5 cohort met histologic criteria for non-alcoholic steatohepatitis (NASH) (S1 Table). There was a non-statistically higher proportion of NAS ≥ 5 patients with stage 1 fibrosis compared to those with NAS ≤3 (88% vs 52%, p = 0.07). Baseline clinical and demographic data is outlined in Table 1. There were no statistically significant differences in age, sex, or ethnicity across the 3 groups. Not unexpectedly, patients with NAS ≤ 3 or NAS ≥ 5 had higher BMI (39.0 ± 8.7 and 34.7 ± 4.1, respectively) than healthy controls (26.3 ±3.5).

**Table 1:** Baseline demographic and clinical characteristics.

	Normal	NAS ≤3	NAS ≥5	P-value
Age (years)	42.9 ± 8.0	47.1 ± 12.6	43.7 ± 9.1	0.187
Sex (%)				
Female	12. (57)	12 (52)	12 (71)	0.492
Male	9 (43)	11 (48)	5 (29)	
Ethnicity (%)				
Non-Hispanic White	18 (86)	18 (78)	13 (77)	
Hispanic White	3 (14)	3 (18)	4 (23)	0.750
Other	0 —	1 (4)	0 —	
***Metabolic Risk Factors***				
BMI	26.3 ± 3.5	39.0 ± 8.7	34.7 ± 4.1	<0.0001
Diabetes (%)	0 —	9 (39)	2 (12)	0.001
On insulin	0 —	4 (17)	1 (6)	0.117
Hyperlipidemia (%)	3 (14)	9 (39)	5 (29)	0.182
On statin	3 (14)	3 (13)	2 (12)	0.999
Hypertension (%)	0 —	14 (61)	6 (35)	<0.0001
OSA (%)	0 —	3 (13)	4 (24)	0.062
Hypothyroidism (%)	1 (5)	5 (22)	3 (18)	0.276
PCOS (%)	0 —	0 —	0 —	0.999
History of CVD (%)	0 —	0 —	1 (6)	0.279
***Laboratory Data***				
AST	18 [16–22.5]	34 [26–45]	66 [47–76]	<0.0001
ALT	19 [13.5–28.5]	46 [33–66]	97 [71–123]	<0.0001
Alk Phos	60 [47–72]	73 [63–95]	77 [60.5–101.5]	0.003
Total Bilirubin	0.7 [0.5–0.8]	0.6 [0.5–0.8]	0.6 [0.5–0.9]	0.766
Albumin	4.3 [4.1–4.5]	4.0 [3.8–4.2]	4.3 [3.9–4.4]	0.041

Baseline demographic, clinical, and laboratory data at time of liver biopsy is shown for each of the three cohorts. Age and BMI are shown as mean ± SD and laboratory data as median [IQR]. Differences across groups were assessed using Kruskal-Wallis test for continuous variables and Chi-square or Fischer’s exact test for categorical variables.

Compared to controls, there was also an increase in prevalence of hypertension and diabetes mellitus in both the NAS ≤3 and NAS ≥ 5 cohorts. There was no difference in prevalence of hyperlipidemia noting three healthy controls were on statin therapy for hyperlipidemia. No other statistically significant differences in NAFLD risk factors were noted across the three groups. Aminotransferases were higher in both NAS≤3 and NAS≥5 patients compared to controls, as was alkaline phosphatase.

### Hepatic transcriptional profiles in NAS ≤3 and NAS ≥5 versus controls

**Fig 1:**
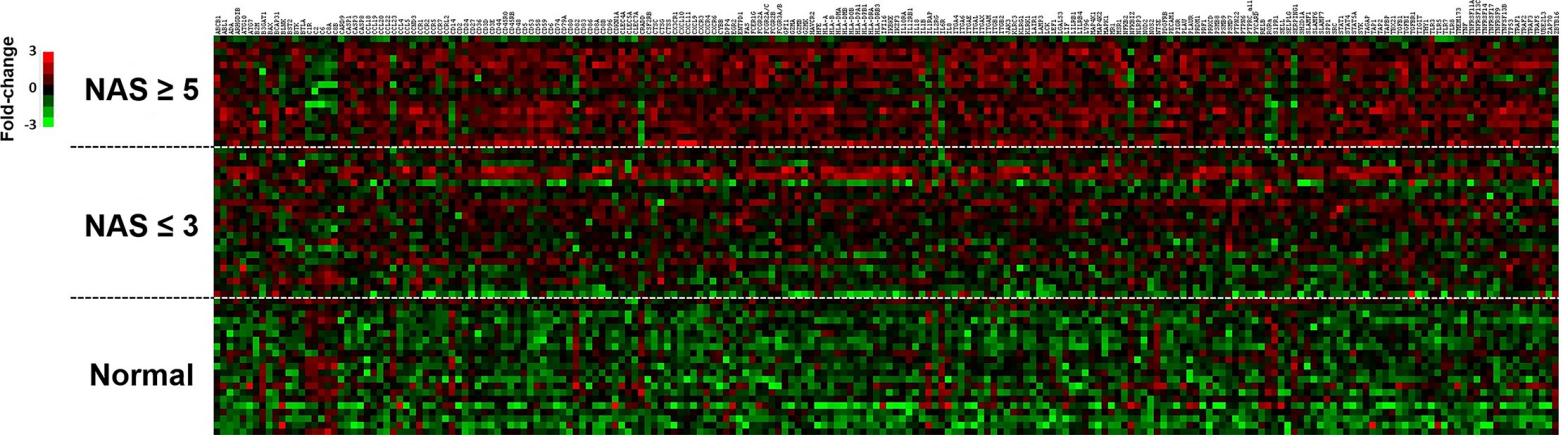
Differential hepatic gene expression in NAFLD progression. Heat map representing color-coded expression levels of differentially expressed genes (FDR<0.01, p<0.01) for each individual sample across the three cohorts (normal, NAS ≤3, and NAS ≥5). A total of 211 genes are included in the heatmap. Z-score transformation was performed with Euclidean distance metric and average expression linkage for bottom-up hierarchical agglomerative clustering using nSolver^®^ Analysis Software v2.6. Red indicates increased relative expression and green decreased relative expression.

**Fig 2:**
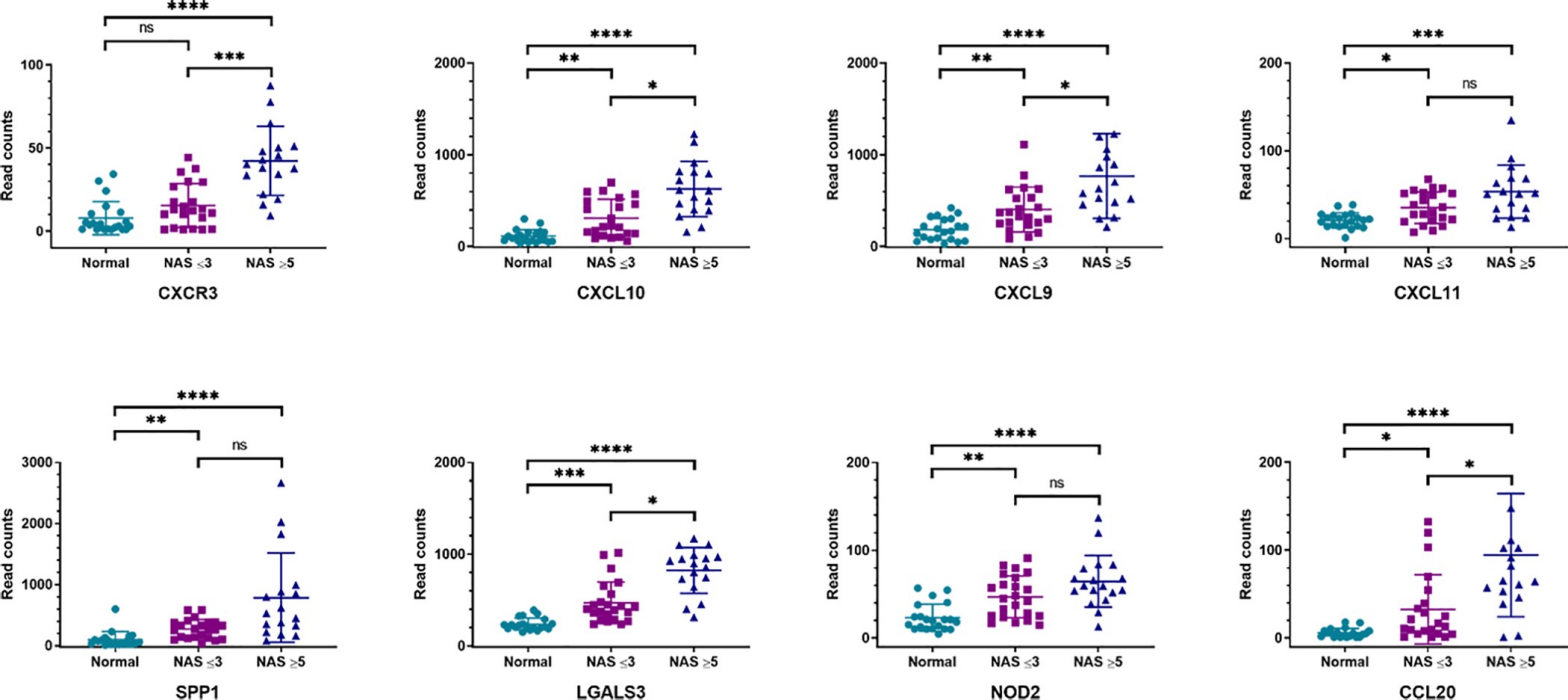
Representative transcriptional differences show stepwise increases across study groups. Individual hepatic gene expression read count comparison in normal (n = 21), NAS ≤3 (n = 23), and NAS ≥5 (n = 18) cohorts. Differences in gene expression across groups was analyzed using Kruskal-Wallis ANOVA with subsequent Dunn’s multiple comparison’s test for pairwise analysis. (* = p<0.05; ** = p<0.01, *** = p<0.001, **** = p<0.0001).

A total of 211 genes were differentially expressed in the NAS ≥5 cohort compared to healthy controls (FDR <0.01, p<0.01). Of these, more than half (109) were differentially expressed in the NAS ≤3 cohort compared to controls (Fig 1). NAS ≤ 3 is associated with significant (>1.5-fold, p<0.01) hepatic induction of multiple chemokines (*CCL20*, *CXCL9*, *CXCL10*, and *CXCL11*); both *SPP1* and *NOD2*, previously been implicated in macrophage activation and M1 polarization [12,13], are also upregulated as compared to controls (S2 Table). In the transition from NAS ≤ 3 to NAS ≥ 5 corresponding to increasing histologic activity, 38 genes were differentially expressed (>1.5-fold, p<0.01) (S3 Table). Of the top 15 differentially expressed genes, *CXCR3*, the receptor for CXCL10, was the most significantly increased in the NAS ≥ 5 cohort (3.74-fold, p<0.001) (Table 2). Notably, hepatic *CCL20*, *CXCL10*, *CXCL8 (IL-8)*, *SPP1*, and *LGALS3* were also significantly upregulated. Fig 2 shows the relative expression of top differentially expressed genes in the three groups. Genes of interest were selected based on the top 5 differentially expressed genes for NAS ≤ 3 (CCL20, SPP1, CXCL10, NOD2, CXCL11) and NAS ≥ 5 (CCL20, CXCR3, SPP1, CXCL10, CXCL9) compared to controls. In addition, given the importance of LGALS3 expression in NAFLD pathogenesis, LGALS3 gene expression was also included. While two genes, PBPP (CXCL7) and MIF, were downregulated in NAS ≥ 5 compared to NAS ≤ 3, there was no difference in gene expression in the individual cohorts compared to healthy controls.

**Table 2:** Top 15 genes differentially expressed between NAS ≥5 and NAS ≤3 cohorts.

Gene	Fold-change	P-value
CXCR3	3.74	0.0002
CCL20	3.69	0.0058
LAMP3	2.87	0.0043
SPP1	2.75	0.0009
CCL22	2.57	0.0018
THY1	2.35	0.0013
CXCL10	2.35	0.0014
IL8	2.31	0.0010
TNFRSF9	2.27	0.0040
EGR2	2.11	0.0066
TNFRSF11A	2.11	0.0074
CLEC5A	2.06	0.0044
LGALS3	1.86	<0.0001
CCL18	1.83	0.0055
RELB	1.8	0.0087

The top 15 statistically significant differences (FDR <0.01, p<0.01) in gene expression between the NAS ≥5 and NAS ≤3 cohorts are shown as relative fold-changes with corresponding p-value. Normalization of gene expression was performed using background subtraction and normalization of gene expression using the geometric mean of 20 housekeeping genes was performed using nSolver^®^ Analysis Software v2.6.

### Plasma levels of gene products using an independent cohort

**Fig 3:**
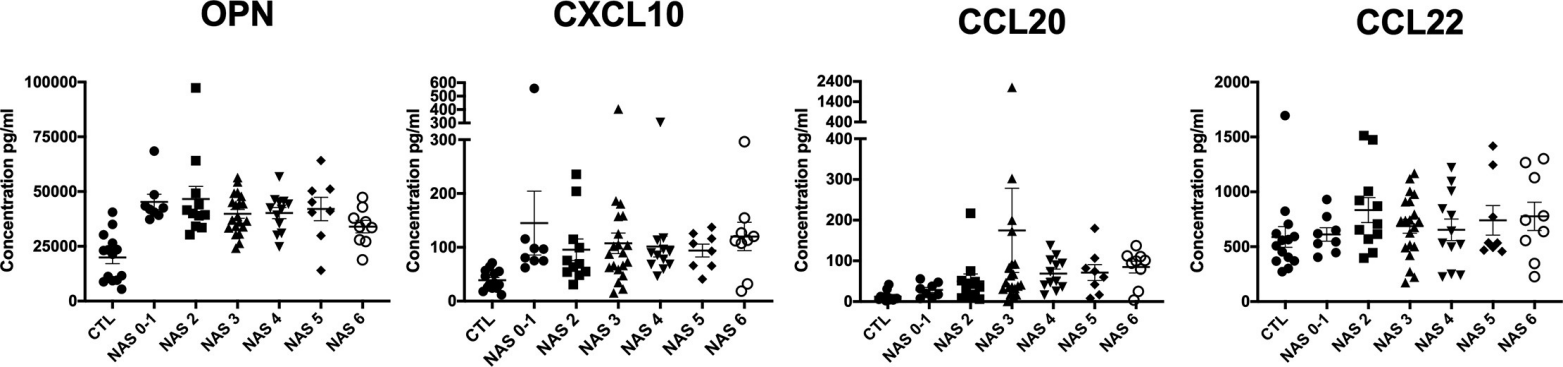
Plasma levels of osteopontin (SPP1) and several chemokines in normal control subjects (n = 14) and patients with NAFLD (n = 67). P < 0.0001 comparing normal to NAFLD groups for OPN and CXCL10.

Next, we sought to determine whether circulating levels of proteins encoded by the genes identified through the transcriptional analysis of hepatic tissue would provide useful information to segregate NAFLD severity. We selected proteins for analysis from those differentially expressed in our cohort with a supported or biologically plausible role in NAFLD pathogenesis. Briefly, using a multiplex assay, plasma samples from 67 patients with varying degrees of biopsy-proven NAFLD (recruited from Virginia Commonwealth) and 15 normal controls were tested. As shown in Fig 3, osteopontin (encoded by *SPP1*) and CXCL10 are both increased in the plasma of subjects with NAFLD, regardless of severity, including those with NAS 0–1. CXCL9 and IL-8 levels were not detected in the vast majority of subjects with NAFLD. IL-8 was only detected in plasma from 2 (14.29%) of the control subjects and 22 (32.84%) of the NAFLD subjects. CXCL9 was not detected in any control plasma and only in 11 of the NAFLD subjects (16.42%).

**Fig 4:**
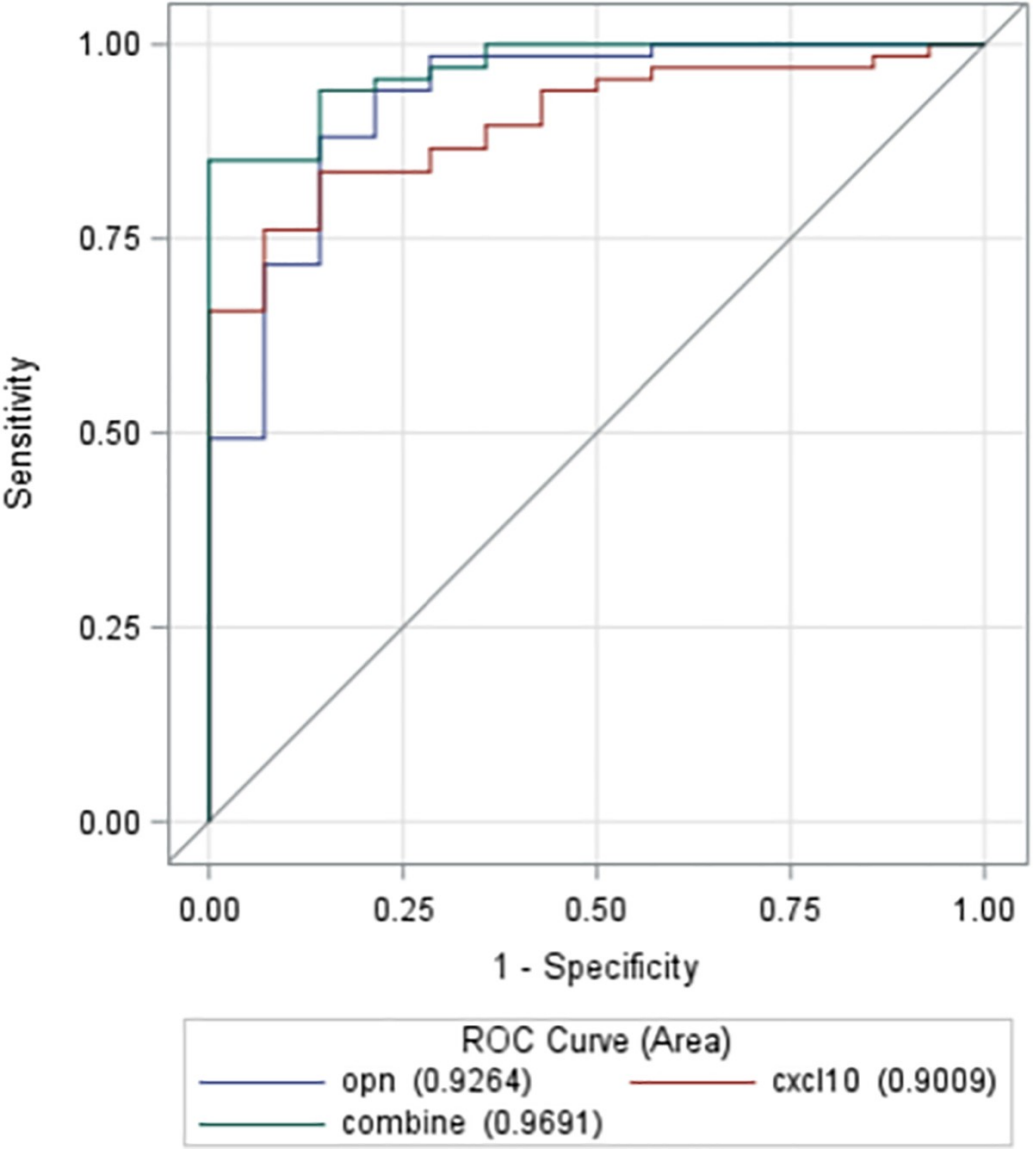
Receiver operating characteristic (ROC) analysis for plasma OPN and CXCL10 levels demonstrate diagnostic accuracy in the prediction of NAFLD. The model has been adjusted for both age and sex.

The elevated plasma levels of OPN and CXCL10 occur early in NAFLD and do not distinguish between NAS<3 and NAS>5 but remain elevated with disease progression. Therefore, we used receiver operating characteristic (ROC) regression to calculate the area under the ROC curve for OPN and CXCL10 separately and in combination to investigate the diagnostic accuracy of OPN and CXCL10 in the prediction of NAFLD. As shown in Fig 4, using a cutoff of 29.88 ng/ml for OPN and 47.63 pg/ml for CXCL10, they do distinguish clearly between the presence or absence of NAFLD when adjusted for both age and gender and may represent a useful diagnostic tool to augment AST/ALT tests in subjects with mild NAFLD enabling early diagnosis and intervention.

## Discussion

The lack of evidence-based, FDA-approved treatment options for NASH underscores the need for further research into understanding disease pathogenesis to identify potential targets [14]. The current study identifies genes that might be pathogenically relevant to development of NAFLD and disease progression. NASH is an inflammatory process largely driven by sterile inflammation and aseptic necrosis [7]. Pro-inflammatory cytokines and chemokines lead to the recruitment of immune cells and perpetuation of liver injury in NASH [7]. Chemokines (e.g., CC, CXC) can activate leukocytes through receptors, previously shown to be upregulated in NASH. Accordingly, a dual chemokine receptor 2 and 5 antagonist (cenicriviroc) prevents macrophage trafficking and is under clinical investigation for the treatment of patients with NASH-related fibrosis [15,16] (NCT03028740 and NCT02217475). Here, in non-fibrotic NAFLD, we found that other chemokines and chemokine receptors were differentially upregulated including *CCL20*, *CXCL9*, *CXCL10*, *CXCL11*, *CCL3*, *CCL4*, *and SPP1*, as compared to normal subjects. Notably, the *SPP1* gene encodes osteopontin (OPN) whose secreted isoform has been found to be chemotactic to immune cells [17] and may represent a conserved pro-fibrogenic response to chronic liver injury [18]. OPN has been implicated in various chronic liver diseases including chronic hepatitis and alcoholic liver disease [19]. OPN upregulation during liver injury and fibrosis represents a conserved repair-response, and thus OPN levels may provide a useful biomarker for liver fibrosis in NASH in addition to other chronic liver diseases. OPN-neutralization abrogates the liver progenitor-cell response and fibrogenesis in several mouse models of liver fibrosis demonstrating that modulation of TGF-β and liver progenitor-cell function by OPN is a key factor driving fibrosis in the liver [18]. Accordingly, a recent study from Duke University demonstrated that advanced fibrosis (stage 3–4 vs fibrosis stage 0–1) in NAFLD was associated with upregulation of OPN [20]. In addition to a pro-fibrogenic role for OPN, it also has several pro-inflammatory properties targeting several innate immune cell populations including macrophages, neutrophils and natural killer cells [19]. A recent study in a murine NASH model demonstrated that antibodies targeting OPN not only attenuated fibrosis but also inflammation, suggesting that targeting OPN in the early stages of NAFLD may be effective in preventing the progression to NASH and fibrosis [21].

Our data suggest that circulating OPN in plasma may reflect early changes in NAFLD in the absence of fibrosis and before inflammation as assessed by standard histology becomes overt and underscores its potential utility as a noninvasive marker in NAFLD, as suggested by others [20, 22]. Recently, we found that oxidized LDL upregulates *SPP1* expression in human and murine macrophages and genetic absence of *SPP1* virtually eliminates hepatic inflammation from a diet-induced murine model of NASH [23].

With regards to other specific chemokines, *CCL20*, also known as macrophage inflammatory protein (MIP-3α), is a chemoattractant for immune cells recently shown to be transcriptionally upregulated in the livers of bariatric surgery patients with NASH-related fibrosis [24]. Furthermore, *CCL20* is induced in hepatic stellate cells exposed to physiological levels of fatty acids (palmitic and oleic acid) [24]. We found a stepwise increase in hepatic transcription of *CCL20* from normal controls to NAS ≤ 3 to NAS ≥ 5, Fig 2 (final panel), although plasma levels could not significantly discriminate across a range of NAS severity. In addition to *CCL20*, which recruits immature dendritic cells, we found C-X-C motif chemokine 10 (*CXCL10*) to be upregulated in both NAS ≤ 3 and NAS ≥ 5 relative to normal controls. *CXCL10* is a hepatocyte-derived chemotactic ligand (also secreted from extracellular vesicles of hepatocytes under lipotoxic conditions) [25] and initiator of inflammatory cascades via its cognate receptor C-X-C motif receptor 3 (*CXCR3*) [26]. Circulating CXCL10 appears to increase relatively early in NAFLD and did not increase with disease severity. *CXCR3* is widely expressed on multiple cells of the innate immune system, including hepatic macrophages, dendritic cells, natural killer (NK) cell, NKT cells, and neutrophils [26]. Moreover, mice genetically deficient in *CXCL10* or its cognate receptor *CXCR3* are protected from diet-induced NASH [26]. Thus, CXCL10 may have utility as an early indicator or target to inhibit inflammation in progression of NAFLD. Importantly, while aminotransferase elevations are common in NAFLD, they are poor at identifying patients with NAFLD. In a recent systematic review and metanalysis, alanine aminotransferase (ALT) was found to be normal in a large percentage of patients with NAFLD (25%) [27]. Furthermore, even in patients with biopsy-proven NASH (NAS ≥ 5), elevated ALT performed poorly as a biomarker for NASH (AUROC 0.62) [28]. Therefore, given the high discriminatory function of circulating CXCL10 and OPN levels, these biomarkers may be of value clinically to identify patients early in NAFLD pathogenesis and perhaps considered for screening populations where widespread imaging is not available.

Several limitations of the study are worth considering. It is possible that obesity or other components of the metabolic syndrome *per se* lead to upregulation of the chemokine and osteopontin pathways even without NAFLD and this question is being addressed by our group. In addition, the two NAFLD patient cohorts comprising this study were cross-sectional; clearly, a longitudinal cohort, including those with weight loss and reversal of NAFLD could provide meaningful information. Taken together, the findings of our pilot study indicate innate immune dysregulation occurs early in NAFLD development (perhaps pre-dating significant histologic inflammation) and that steatosis and steatohepatitis represent different stages in the inflammatory evolution of NAFLD, in keeping with the recent recognition that progression of fibrosis can occur even in patients with bland steatosis on the index biopsy [29]. These considerations are important as we search to identify and develop novel biomarkers for this common disease.

## Supporting information

S1 Table NAFLD activity score analysis. Individual histologic criteria (steatosis, ballooning hepatocytes, lobular inflammation) are described for the NAS ≤3 and NAS ≥5 cohorts. Mean score is shown for each individual category as well as summative NAS score. There were statistically significant differences in each individual category as well as summative NAFLD activity score.
(DOCX)

S2 Table Fold-change in gene expression (NAS ≤3 vs normal). Statistically significant differences (FDR <0.01, p<0.01) in gene expression between the NAS ≤3 and Normal cohorts are shown as relative fold-changes with corresponding p-value. A total of 14 genes had >1.5-fold differentially expression between the groups. Normalization of gene expression was performed using background subtraction and normalization of gene expression using the geometric mean of 20 housekeeping genes was performed using nSolver^®^ Analysis Software v2.6.
(DOCX)

S3 Table Fold-change in gene expression (NAS ≥5 vs NAS ≤3). Statistically significant differences (FDR <0.01, p<0.01) in gene expression between the NAS ≥5 and NAS ≤3 cohorts are shown as relative fold-changes with corresponding p-value. A total of 37 genes were differentially expressed between the groups. Normalization of gene expression was performed using background subtraction and normalization of gene expression using the geometric mean of 20 housekeeping genes was performed using nSolver^®^ Analysis Software v2.6.
(DOCX)
