# Supplementary material for: Increased hepatic and circulating chemokine and osteopontin expression occurs early in human NAFLD development
Source: PLoS One. 2020 Jul 30;15(7):e0236353. doi: 10.1371/journal.pone.0236353 (PMC7392333; doi:10.1371/journal.pone.0236353)

**S1 Table. NAFLD Activity Score Analysis.**

Individual histologic criteria (steatosis, ballooning hepatocytes, lobular inflammation) are described for the NAS ≤3 and NAS ≥5 cohorts. Mean score is shown for each individual category as well as summative NAS score. There were statistically significant differences in each individual category as well as summative NAFLD activity score.


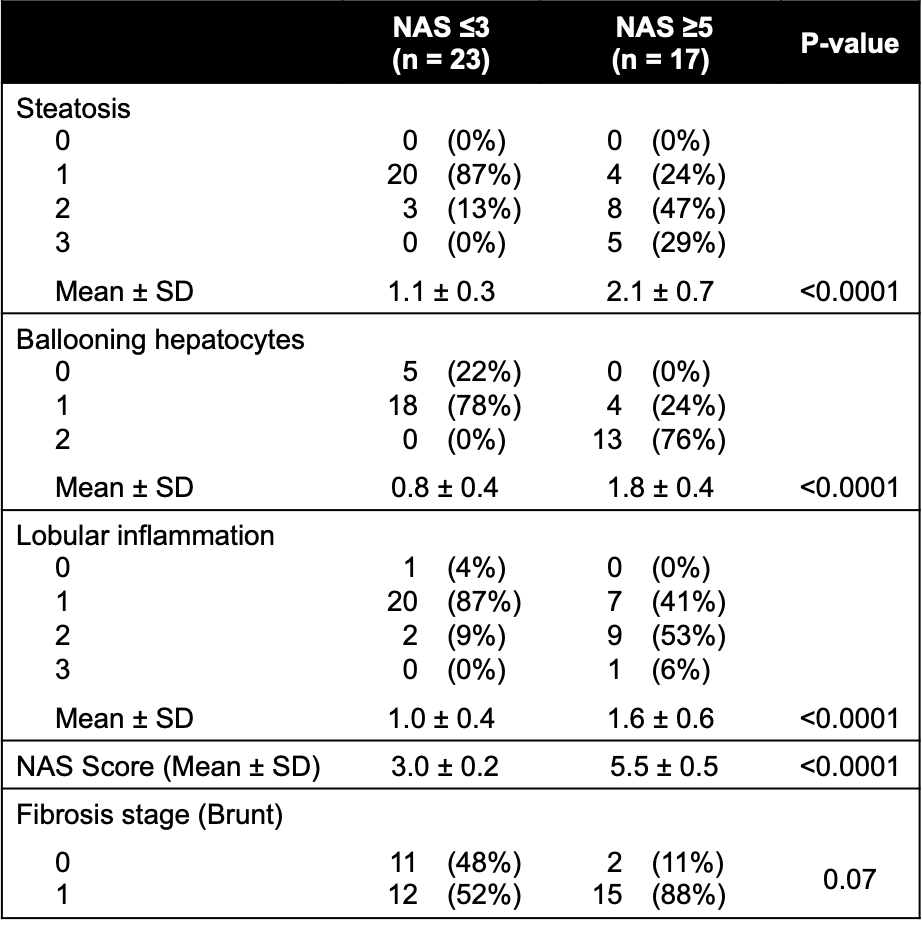

Supplement: S1 Table — Individual histologic criteria (steatosis, ballooning hepatocytes, lobular inflammation) are described for the NAS ≤3 and NAS ≥5 cohorts. Mean score is shown for each individual category as well as summative NAS score. There were statistically significant differences in each individual category as well as summative NAFLD activity score. (DOCX) [file pone.0236353.s001.docx]
