# Supplementary material for: Increased hepatic and circulating chemokine and osteopontin expression occurs early in human NAFLD development
Source: PLoS One. 2020 Jul 30;15(7):e0236353. doi: 10.1371/journal.pone.0236353 (PMC7392333; doi:10.1371/journal.pone.0236353)
Supplement: S2 Table — Statistically significant differences (FDR <0.01, p<0.01) in gene expression between the NAS ≤3 and Normal cohorts are shown as relative fold-changes with corresponding p-value. A total of 14 genes had >1.5-fold differentially expression between the groups. Normalization of gene expression was performed using background subtraction and normalization of gene expression using the geometric mean of 20 housekeeping genes was performed using nSolver® Analysis Software v2.6. (DOCX) [file pone.0236353.s002.docx]

**S2 Table.** **Fold-Change in Gene expression (NAS ≤3 vs Normal).**

Statistically significant differences (FDR <0.01, p<0.01) in gene expression between the NAS ≤3 and Normal cohorts are shown as relative fold-changes with corresponding p-value. A total of 14 genes had >1.5-fold differentially expression between the groups. Normalization of gene expression was performed using background subtraction and normalization of gene expression using the geometric mean of 20 housekeeping genes was performed using nSolver^®^ Analysis Software v2.6.

**
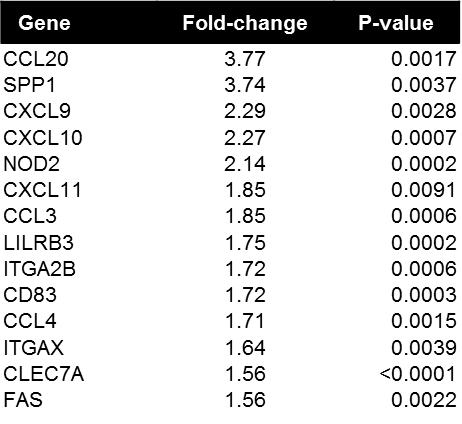
**
